# Establishing an Independent Mobile Health Program for Chronic Disease Self-Management Support in Bolivia

**DOI:** 10.3389/fpubh.2014.00095

**Published:** 2014-08-13

**Authors:** John D. Piette, Helen Valverde, Nicolle Marinec, Rachel Jantz, Kevin Kamis, Carlos Lazo de la Vega, Timothy Woolley, Bismarck Pinto

**Affiliations:** ^1^Center for Clinical Management Research, VA Ann Arbor Healthcare System, Ann Arbor, MI, USA; ^2^University of Michigan School of Public Health, Ann Arbor, MI, USA; ^3^University of Michigan School of Medicine, Ann Arbor, MI, USA; ^4^Servicio Departamental de Salud, La Paz, Bolivia; ^5^Universidad Católica Boliviana, La Paz, Bolivia; ^6^IEQ Technology, Inc., Springfield, OR, USA

**Keywords:** mobile health, disease management, chronic illness, vulnerable populations, Latin America

## Abstract

**Background:** Mobile health (m-health) work in low- and middle-income countries (LMICs) mainly consists of small pilot programs with an unclear path to scaling and dissemination. We describe the deployment and testing of an m-health platform for non-communicable disease (NCD) self-management support in Bolivia.

**Methods:** Three hundred sixty-four primary care patients in La Paz with diabetes or hypertension completed surveys about their use of mobile phones, health and access to care. One hundred sixty-five of those patients then participated in a 12-week demonstration of automated telephone monitoring and self-management support. Weekly interactive voice response (IVR) calls were made from a platform established at a university in La Paz, under the direction of the regional health ministry.

**Results:** Thirty-seven percent of survey respondents spoke indigenous languages at home and 38% had six or fewer years of education. Eighty-two percent had a mobile phone, 45% used text messaging with a standard phone, and 9% had a smartphone. Smartphones were least common among patients who were older, spoke indigenous languages, or had less education. IVR program participants completed 1007 self-management support calls with an overall response rate of 51%. IVR call completion was lower among older adults, but was not related to patients’ ethnicity, health status, or healthcare access. IVR health and self-care reports were consistent with information reported during in-person baseline interviews. Patients’ likelihood of reporting excellent, very good, or good health (versus fair or poor health) via IVR increased during program participation and was associated with better medication adherence. Patients completing follow-up interviews were satisfied with the program, with 19/20 (95%) reporting that they would recommend it to a friend.

**Conclusion:** By collaborating with LMICs, m-health programs can be transferred from higher-resource centers to LMICs and implemented in ways that improve access to self-management support among people with NCDs.

## Introduction

### m-Health in low and middle-income countries

Most people die from non-communicable diseases (NCDs), 80% of which occur in low- and middle-income countries (LMICs) where mortality rates are twice those of industrialized nations (1–4). The years of life lost to diabetes increased by 29% from 2000 to 2010 and the global prevalence of diabetes will be 439 million in 2030 (5, 6). More than one in four adults worldwide has hypertension, with two-thirds living in LMICs (7). In Latin America, more than 100 million adults are hypertensive, with rates among the highest in the world (7). Hypertension is the main contributor to the disease burden globally and the leading cause of cardiovascular diseases (1, 2, 8). Most adults with hypertension and other NCDs rely on primary care for disease management. However, LMICs still have weak primary care systems that lack capacity and resources to help patients effectively treat these conditions.

In the face of these resource constraints, models for developing mobile health (m-health) tools to improve patient monitoring and self-care support are especially important. Mobile phones are ubiquitous in LMICs (9–12), and because m-health services have low marginal costs and high availability, they have the potential to reach large numbers of patients between in-person clinical encounters. Many studies have demonstrated that m-health services including short-message service (SMS or text messaging) and interactive voice response (IVR) calls can improve NCD self-care support. Randomized trials suggest that m-health interventions can improve outcomes of chronic illness care in LMICs as well as a range of important self-care behaviors (13–17). Studies in Africa have demonstrated that SMS can improve patients’ medication adherence (18, 19), while SMS messages to health workers can improve the quality of disease management (16). SMS studies in other countries also have had promising results (20–22). Monitoring and self-care support using IVR can complement SMS and provide a scalable solution to: collect complex clinical information; deliver more complex self-care education messages; and communicate with patients who have limited literacy, vision, or dexterity for texting. However, the challenge that has frustrated the field of m-health has been termed “pilot-itis,” i.e., the repeated conduct of small, short-term, and non-sustainable pilot programs without a clear model for disseminating approaches from expert centers or scaling them in order to address the needs of large populations.

We have developed potentially sustainable models for m-health self-management support for vulnerable populations in the US and Latin America (23–30). In 2010, we collaborated with community leaders in Honduras to evaluate the feasibility of delivering m-health self-management support to patients with diabetes in that country via IVR and a cloud-computing approach (31). Patients received weekly monitoring and self-management support calls, during which they reported clinical information and received tailored self-management education. Based on those reports, clinicians received automated notifications via email identifying patients needing additional assistance. At follow-up, participants reported high levels of intervention satisfaction and improvements to self-care behaviors, and glycemic control improved significantly. In 2011, we conducted a randomized trial evaluating the efficacy of m-health hypertension management among patients in Honduras and Mexico (32). Intervention patients’ systolic blood pressures improved and patients reported fewer depressive symptoms, fewer medication problems, better general health, and greater satisfaction with care (32).

### Developing m-health programs for NCD management in Bolivia

Bolivia is one of the most economically challenged countries in the Western Hemisphere. It has a population of 10.5 million with 51% living at or below the poverty line (33). Thirty-five percent of Bolivians live in rural areas. Bolivia’s health system includes both public and private providers of “western” medicine, and traditional medical providers serve 10% of the population (34).

Since passage of national reforms in 2007, all Bolivians have a legal right to access health care. Publically provided services are available in some areas for specific population subgroups, such as the elderly, pregnant women, and infants. However, economic, geographic, cultural, and social barriers limit access to health care for 77% of the population (35). The majority of the employed population (69%) works in the informal labor market, and thus is not eligible for most public or private health insurance programs (34). In 2009, 57% of all Bolivians were without any health insurance coverage (36) and only 12% are able to pay for private health insurance (36). Even when insurance is available, supply and infrastructure constraints limit healthcare access (36, 37). Bolivia has 1.2 doctors and 2.1 nurses per 1000 inhabitants (36), and of the 2875 primary care centers, 45% do not have a physician (2007 data) (35).

Since 2012, we have worked in collaboration with the Institute of Applied Engineering at la Universidad Católica Boliviana in La Paz to package the IVR platform, developed at the University of Michigan, for transfer and installation as an independent program in La Paz. Beginning in 2013, we initiated a multi-year project to better understand the ways in which m-health interventions could assist Bolivian health officials and clinicians in addressing the needs of the population. Here, we describe the results of a survey designed to provide information about the potential reach and scalability of m-health services for NCD self-care support in Bolivia as well as the results of initial testing of the IVR platform among patients with diabetes and/or hypertension.

## Materials and Methods

### Organizational partners

The primary organizational partners for this study were: the program on Quality Improvement for Complex Chronic Conditions at the University of Michigan (UM); the NCD epidemiology unit in el Servicio Departamental de Salud (SEDES or Departmental Health Services) in La Paz, Bolivia, and la Universidad Católica Boliviana (UCB or Bolivian Catholic University), also in La Paz. Within UCB, the project was jointly directed by the Institute for Research on Behavioral Science and the Institute for Applied Engineering, both working under the direction of the Office of the Rector. Two other universities in La Paz – la Universidad Mayor de San Andrés and la Universidad Pública de El Alto – also played important roles as facilitators and collaborators in survey data collection.

### Cross-sectional quantitative survey

In order to understand NCD patients’ needs for m-health support and their access to mobile phones, we conducted a face-to-face cross-sectional survey between June and August 2013. Under the direction of SEDES, potential participants were approached at the time of visits to: primary care clinics in two public hospitals and one private hospital in La Paz, one public hospital in El Alto, and two hospital-sponsored health fairs. Surveys were administered by a team of UM graduate students fluent in Spanish, working in collaboration with Bolivian clinicians and medical students. Native Spanish speakers reviewed and tested the survey prior to patient recruitment. The study was approved by both UM and UCB ethics committees and all participants provided written informed consent.

A total of 1144 patients aged 18 years or older completed the initial survey module. This module captured information about respondents’ age, household income, occupation, educational attainment, marital status, reasons for their visit to the clinic or health fair, NCD diagnoses, medication use, social support, and access and utilization of mobile phones. A total of 664 participants also completed a second, more extensive survey module. Patients were eligible for this second module if they: reported having one or more diagnosed chronic conditions, had systolic blood pressures indicating potential hypertension (systolic pressures >140 mmHg), or scored positive on the Patient Health Questionnaire (PHQ) two-item depression screener (38). The more extensive survey included questions about respondents’: access and utilization of health care services, alcohol use [as measured by the Alcohol Use Disorders Identification Test or AUDIT (39)], tobacco use, NCD self-care, blood pressure self-monitoring (for hypertension patients), glucose self-monitoring (for diabetes patients), perceptions about the quality of health services [as measured by the Patient Assessment of Chronic Illness Care or PACIC scale (40)], depressive symptoms [as measured by the PHQ-8 (41)], ethnicity, and language spoken at home. Patients who completed the survey were given a small gift that included toiletries such as toothbrushes, toothpaste, and lip balm. The data presented here are limited to the subsample of 364 patients who completed both survey modules and were potentially eligible for the IVR pilot study, i.e., patients who reported either a diabetes or hypertension diagnosis, or who had a systolic blood pressure at the time of the survey of >140 mmHg.

### IVR trial

One hundred sixty-seven patients with diabetes and/or hypertension were approached for enrollment in the trial of the IVR platform. Two patients were unable to complete the enrollment because of time constraints, and the remaining 165 were enrolled. Technical details about the IVR platform, including the hardware and software requirements, are available from the authors on request. In addition to weekly IVR calls as described below, patients with hypertension received a home blood pressure monitor and were trained in its use, including how to keep a daily record of their systolic blood pressure values. All participants received 12 weekly IVR calls to their mobile or landline telephones, with multiple call attempts at times the patient indicated were convenient. Calls emanated from the m-health platform established at UCB and the system used a GSM gateway and SIM cards to make calls directly to local mobile phone towers. The IVR script used a tree-structured algorithm to assess patients’ self-management behaviors, perceived health status, and symptoms. The call script was initially developed in English with input from experts in NCD management, primary care, and m-health. The script was then professionally translated into Spanish and reviewed by Bolivian primary care providers for cultural and linguistic appropriateness. Quality assurance testing was performed on all IVR programming, and website interface prototypes were evaluated by clinicians and staff in each location using free exploration and performance tests to ensure appropriate navigation and system utilization (42).

The content and structure of the IVR interactions in both English and Spanish are available as Supplementary Material. In brief, during each call, patients reported information about their perceived health status (excellent, very good, good, fair, or poor); changes in their health compared to the prior week; diabetes and antihypertensive medication adherence; whether the patient had enough medication to last 2 weeks; (for hypertensive patients) whether they or someone else was regularly checking their blood pressure at home; (if checking) whether they regularly had high systolic blood pressure readings (i.e., >130 mmHg if diabetic or >140 mmHg if non-diabetic), whether they regularly had systolic blood pressure readings <100 mmHg, and whether they were avoiding foods high in salt. Based on patients’ responses, they were given tailored self-management education and, as needed, were advised to contact their doctor. For example, if a patient with both diabetes and hypertension reported regularly experiencing systolic blood pressures >130 mmHg, he or she was told (in Spanish) the following:

Your blood pressure may be too high. Even blood pressure that is just a little higher than normal can worsen the complications of diabetes. If you cut back on your salt intake, you may be able to get your blood pressure level down. However, many people need to adjust their medication to bring their blood pressure in line. It is important that you make an appointment with your doctor soon so that you can tell your doctor that your blood pressure has been running higher than normal.

At each participating clinic, we identified a primary physician who served as the point of contact for receiving and acting on structured email notifications generated automatically based on patients’ IVR health and self-care reports. Notifications included the patient’s full name, and the time and date that the patient reported the information generating the alert. For patients with diabetes, clinician alerts were generated if the patient reported rarely or never taking their antihyperglycemic medication as prescribed or (if also hypertensive) the patient reported systolic blood pressure readings >130 or <100 mmHg on two or more days in the prior week. For patients with hypertension, clinical alerts were generated if the patient reported rarely or never taking their antihypertensive medication as prescribed or reported regularly experiencing systolic blood pressures >140 or <100 mmHg on two or more days in the prior week.

### Qualitative follow-up interviews

We identified a purposive sample of 20 patients who participated in the IVR program for follow-up semi-structured telephone interviews. Patients were contacted via their mobile phone and all interviews were conducted by bilingual research associates. Patients’ responses were documented using a semi-structured form. The interviews were not audio-recorded. We sampled patients from each recruitment site to represent diverse experiences, including patients with varying numbers of completed IVR calls, both genders, and a mixture of patients with diabetes and hypertension. Questions addressed themes such as patients’ overall satisfaction with the program, whether or not the patient felt that the program helped their chronic disease self-management, and whether the patient would recommend the program to a friend or relative if that person also were diagnosed with one of the target health conditions. Respondents were also asked, “If the program were available in your clinic, would you use it again?” Out of the 37 patients initially identified, 20 completed telephone interviews, 15 could not be contacted by phone, and 2 declined participation.

### Analysis

Analyses of the quantitative survey data focused on the bivariate relationships between patients’ sociodemographic characteristics (age, gender, ethnicity, education, and need for health information), clinical characteristics (self-reported general health status and number of reported chronic diseases), and health care access; and, their access and use of m-health technology. As a proxy for indigenous ethnicity, we identified patients who reported speaking an indigenous language (i.e., a language other than Spanish, such as Aymara) in the home. High health information need was defined as self-reported illiteracy or “frequently” or “always” needing help understanding health information, as defined by a widely used screener (43). Patients with healthcare access problems were defined as those reporting that cost kept them from going to the clinic or hospital in the past year (a measure of financial access) and patients who had longer travel times to clinic (a measure of geographical access). Patients’ access and use of m-health technology was characterized using a four-level hierarchical variable identifying patients without a mobile phone; patients who had a standard mobile phone but who were unable to text; patients with a standard mobile phone who used texting; and patients with a smartphone.

The primary outcome for the IVR demonstration was patient engagement defined as “call completion.” We examined engagement using a dataset including one record for each week in which a contact was attempted (i.e., approximately 12 weeks per participant). We created a merged dataset linking each call week of experience to the patient’s baseline survey reports and we used logistic regression to identify the patient characteristics associated with call completion. In order to ensure that the system did not automatically re-call patients who hung up early, calls were considered completed after the patient responded to the first general health question. In practice, patients who accepted an IVR call almost always stayed connected until the end of the call. Predictors of call completion for the multivariate model represented each of the patient characteristics shown in Table 1, and we used cubic splines and a graphical display to describe the variation in IVR call engagement across age groups controlling for covariates. To evaluate the validity of patients’ IVR clinical reports, we examined the correlation between: (1) IVR health and self-care reports and (2) patients’ perceived health status and medication adherence reported at baseline. Medication adherence at baseline was measured using a validated adherence scale (44). Finally, we used multivariate logistic regression (controlling for patient characteristics shown in Table 1) and patients’ IVR reports of perceived health status to characterize changes in health status over the course of patients’ 12-week participation in the program. Specifically, we constructed a model estimating the probability that the patient would report excellent, very good, or good health during an IVR call (versus fair or poor health), with call week being the predictor of interest and controlling for baseline patient characteristics. To shed light on observed changes in patients’ IVR-reported perceived health over time, we examined the correlation between IVR health status reports and patients’ likelihood of reporting taking their medication as prescribed during the same call. All analyses of call week-level data were conducted taking into account the clustering of calls within patient.

**Table 1:** **Characteristics of primary care patients with diabetes and/or hypertension in La Paz, Bolivia by level of engagement with mobile technology**.

*N* = 364	*N* (%)^e^	Level of technology use^f^				*P*-value
		Level 0	Level 1	Level 2	Level 3
Total	364 (100)	17.7	28.5	45.0	8.8	
**Demographics**
Age
18–29	14 (3.9)	14.3	14.3	50.0	21.4	<0.0001
30–49	60 (16.5)	10.0	20.0	55.0	15.0	
50–65	163 (44.8)	8.0	30.9	51.9	9.3	
65+	127 (34.9)	34.1	31.0	31.0	4.0	
Gender
Male	150 (41.2)	9.4	26.9	53.7	10.1	0.0021
Female	214 (58.8)	23.5	29.6	39.0	8.0	
Indigenous language at home
Yes	133 (36.5)	16.7	38.6	39.4	5.3	0.0071
No	231 (63.5)	18.3	22.6	48.3	10.9	
Education in years
6 Or less	133 (38.1)	26.3	43.6	24.8	5.3	<0.0001
7–12	132 (37.8)	13.7	24.4	54.2	7.6	
More than 12	84 (24.1)	9.6	8.4	63.9	18.1	
High information needs^a^
Yes	139 (38.2)	29.0	36.2	29.7	5.1	<0.0001
No	225 (61.81)	10.7	23.7	54.5	11.2	
**Health status**
Fair/poor perceived health
Yes	252 (70.2)	16.0	30.0	45.2	8.8	0.5949
No	107 (29.8)	21.5	25.2	44.9	8.4	
No. of chronic conditions^b^
0	7 (2.0)	14.3	28.6	57.1	0.0	0.9262
1	128 (36.3)	14.3	27.8	47.6	10.3	
≥2	218 (61.8)	18.8	28.9	44.0	8.3	
Diabetes and hypertension^c^
Hypertension only	163 (44.8)	16.1	27.2	46.3	10.5	0.8518
Diabetes only	94 (25.8)	21.5	30.1	40.9	7.5	
Both conditions	107 (29.4)	16.8	29.0	46.7	7.5	
**Health care access**
Cost barriers^d^
Yes	154 (42.5)	17.1	33.6	41.5	7.9	0.3608
No	208 (57.5)	18.3	25.0	47.6	9.1	
Travel time to clinic
0–29 min	136 (40.2)	24.3	23.5	42.7	9.6	0.0516
30–59 min	121 (35.8)	15.1	26.9	49.6	8.4	
≥60 min	81 (24.0)	11.1	40.7	39.5	8.6	

*Cell entries are row percents, except the *N* (%) column, which includes marginal/column percents. *N*’s in some cells represent a small amount of missing data on that survey item*.

*^a^High information needs defined as being illiterate, frequently or always needing someone to help read papers from the health center, or frequently or always having problems understanding written medical instructions*.

*^b^Includes patients’ report of a physician diagnosis of: diabetes, depression, cancer, hypertension, arthritis, chronic back pain, cardiovascular disease (heart attack, blocked artery, or stroke), and pulmonary disease (emphysema, COPD, or asthma)*.

*^c^Self-reported physician diagnosis or measured systolic blood pressure >140 mmHg if hypertensive, or >130 mmHg if diabetic OR hypertensive and diabetic*.

^d^In the past year, did cost ever keep you from going to a clinic or hospital?

*^e^Column percent*.

*^f^Level 0: does not own cell phone; Level 1: owns personal cell phone but unable to text. The cell phone is not a smart phone or is shared; Level 2: owns a cell phone and able to text. Cell phone is not a smart phone; Level 3: owns a smart phone*.

## Results

### Cross-sectional quantitative survey

A total of 1114 primary care patients completed the initial module of the quantitative survey of their demographic characteristics, diagnoses, and mobile phone use. Of these, 664 patients with chronic illnesses completed the more extensive survey of their self-care and service use, including 364 respondents who either reported a diagnosis of diabetes or hypertension, or who had a resting systolic blood pressure reading of 140 mmHg or higher (Table 1). Twenty-six percent had diabetes but not hypertension, 45% had both conditions, and the remaining 29% had hypertension but not diabetes. Patients represented a broad distribution of ages, and most respondents (59%) were women. A total of 37% reported speaking an indigenous language in the home and 38% had no more than 6 years of formal education. Most patients (70%) reported fair or poor health status and 43% reported that they avoided seeking care at least once in the prior year due to the cost. While 40% of patients lived <30 min from their source of primary care, 24% traveled more than an hour, with many of those patients coming from rural areas outside of La Paz.

Overall, 18% of respondents with diabetes or hypertension had no mobile phone, 29% had a standard mobile phone but did not text, 45% had a standard mobile phone and used texting, and only 9% had a smartphone. Patients’ sociodemographic characteristics, health status, and healthcare access were related to their level of engagement with mobile phone technology. For example, while only 14% of respondents between 18 and 29 years of age had no phone, this was true for 34% of respondents over age 65 years. In contrast, 21% of respondents aged 18–29 years had a smartphone – more than five times as many as patients who were aged 65 years or higher (4%). Smartphone access was less common among patients who spoke indigenous languages in the home, had less education, or had greater health information needs.

### IVR trial

A total of 165 patients from the participating primary care centers and health fairs participated in the trial of the Bolivian IVR platform. Twenty-three percent of patients had diabetes without hypertension, 48% had hypertension without diabetes, and the remaining 29% of patients had both conditions. Compared to patients who did not participate, IVR study participants were significantly older on average (60 versus 49 years, *P* < 0.001) but were similar in terms of years of education, gender, and ethnicity.

**Figure 1:**
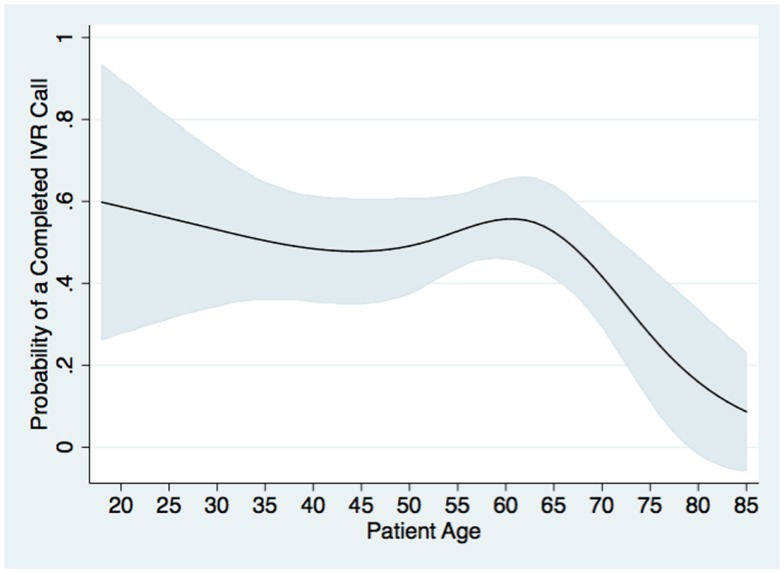
**Probability of completing a given weekly IVR monitoring and self-management support call (*Y* axis) by patient age (*X* axis) is shown**. Probabilities were estimated from a two-level multivariable logistic model in which the outcome of each patient-week of call attempts was analyzed, with call weeks nested within patient, and controlling for patients’ baseline sociodemographic, clinical, and access characteristics as shown in Table 1. Gray bands represent 95% confidence intervals for the predicted probabilities.

Interactive voice response study participants completed 1007 weekly IVR self-management support calls out of 1995 weekly calls attempted, for an overall completion rate of 51%. Thirty percent of patients completed more than nine IVR calls and 15% completed calls for 11 or 12 weeks in which one was attempted. Nineteen percent of participants were never successfully reached. In multivariate analysis, the probability of completing an IVR call was not related to patients’ gender, ethnicity, health status, or self-reported healthcare access. Call completion was significantly associated with patients’ level of educational attainment (*P* = 0.03). Compared to patients with not more than 6 years of schooling, the odds of call completion was 2.4 times as high (95% CI: 1.2, 4.6) among users with >12 years of education and was marginally higher among users with 7–12 years (adjusted odds ratio: 1.4; CI: 0.8, 2.6). The odds of completing an IVR call were 3.0 times higher among patients with hypertension (with or without diabetes) when compared to patients with diabetes alone (*P* < 0.001), possibly due to the fact that hypertension patients received a home blood pressure monitor in addition to the IVR calls. Call completion was roughly constant across age groups until approximately age 65 years, and then declined precipitously, see Figure 1.

**Figure 2:**
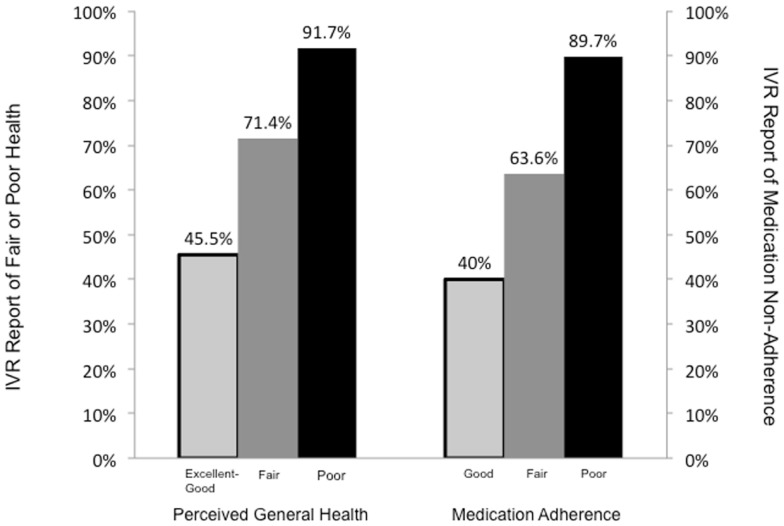
**The proportion of patients who reported (left) fair or poor general health and (right) medication adherence problems at least once via IVR, within groups defined by that patient’s baseline survey reports (*X* axis)**. Baseline medication adherence was measured using the Morisky adherence scale, with scores collapsed into groups as follows: “Good” = 0, “Fair” = 1–2, and “Poor” = 3–8.

Patients’ responses during IVR assessments were associated in expected ways with their health and self-care behaviors reported at baseline, see Figure 2. For example, 92% of patients who reported poor health at baseline reported fair or poor health at least once during an IVR assessment, compared to 71% of patients reporting fair health at baseline, and 45% of patients reporting good, very good, or excellent health at baseline (*P* < 0.001). Patients were also more likely to report problems taking their medication as prescribed during their IVR calls if they had worse medication adherence scores at baseline, reported using the Morisky measure (44).

**Figure 3:**
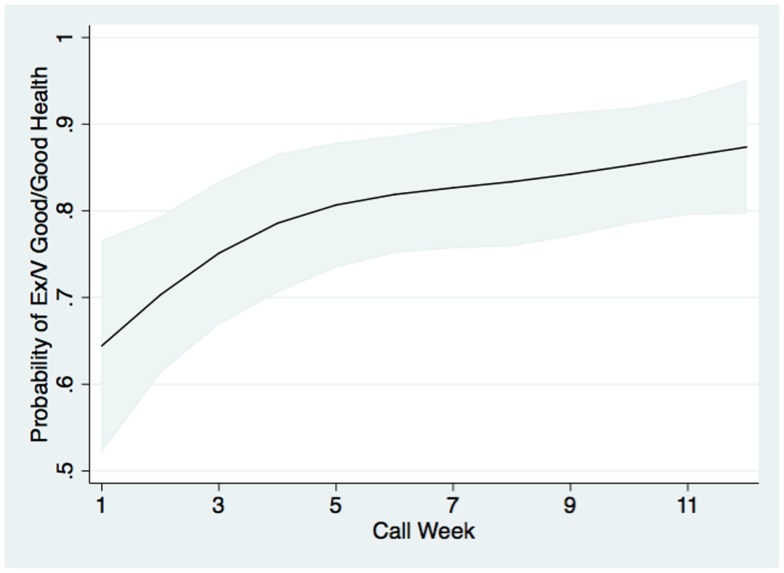
**The probability that patients completing an IVR call on a given week reported excellent, very good, or good health (versus fair or poor health)**. Probabilities were estimated from a two-level multivariable logistic model using data from all IVR pilot study participants, with call weeks nested within patient, and controlling for patients’ sociodemographic, clinical, and access characteristics as shown in Table 1. Gray bands represent 95% confidence intervals for the predicted probabilities.

In multivariate modeling, patients were 3.3 times as likely to report fair or poor health during an IVR call if they reported fair or poor perceived health status during their baseline survey (*P* < 0.0001). They were also more likely to report fair or poor health via IVR if they had fewer years of education, and were 2.2 times as likely to report fair or poor health via IVR if they had greater baseline-reported health information needs (both *P* < 0.01). Patients’ likelihood of reporting excellent, very good, or good health via IVR increased during their participation in the program, from a probability of roughly 64% in week 1 to 88% in week 12, see Figure 3. When patients reported good, very good, or excellent health during an IVR call, during the same call they also were more likely to report that they took their medication exactly as prescribed (*P* < 0.001). Specifically, 61.5% of patients reporting good, very good, or excellent health also reported always taking their medication as prescribed in the past week when compared to 45.9% of patients reporting fair or poor health. In contrast, when patients reported good, very good, or excellent health, they were less than half as likely as patients with fair or poor health to report that they rarely or never took their medications as prescribed in the last week (14.4 versus 29.9%; *P* < 0.001).

Despite these encouraging findings, a large number of completed IVR calls generated clinician alerts. Overall, 52.8% of all completed IVR calls generated a clinician alert, with 20.6% of all completed calls generating an alert for fair or poor medication adherence, and 38.7% of assessments to patients with hypertension generating a clinician alert for high or low systolic blood pressure levels. Fair or poor medication adherence was reported 37.2% of the time among patients with diabetes plus hypertension, 20.3% of the time by patients with hypertension alone, and 15.9% of the time among patients with diabetes but not hypertension.

### Qualitative follow-up interviews

Of the 20 IVR program participants who completed follow-up interviews, nearly all (19/20 or 95%) rated the program as either excellent or very good, and most patients reported that the program satisfied the majority of their needs (84%). Almost all patients reported that they would recommend the program to a friend (19/20 or 95%), and 14 of 20 patients (70%) were very satisfied with the amount of assistance they received via the IVR calls. If the program were offered in the future, 84% (17/20) reported that they would definitely use it again. In open-ended questions, most patients reported that they had learned something from the program, either about measuring their blood pressure, remembering to take their medication, or improving their diet. Several patients (8/20 or 40%) reported being pleased that they were reminded to take their medications. In addition, nine patients (45%) noted that they liked the recommendations for improving their self-care.

When asked about weaknesses of the program, most respondents reported that they could not think of any. However, six patients noted that sometimes the telephone connection made it difficult to hear the recorded messages. Three patients had difficulty in responding to the questions, either because they could not keep up with the speed of the call or because their mobile phone was malfunctioning. Five patients reported that they wished that they could have spoken directly to a healthcare provider. Four patients would have liked to be able to change the days and times on which they received the calls without having to contact the project team, and four patients would have liked more information on topics such as diet and other diseases.

## Discussion

### Summary of findings

Here, we describe our efforts to transfer an m-health platform from a research center in the US to an LMIC under the direction of that country’s health ministry and in collaboration with academic partners in both countries. The project was the first of its kind in Bolivia. Although Bolivia faces significant infrastructure challenges, we found that the establishment of an m-health platform was technically feasible. Most adult patients with diabetes or hypertension recruited in primary care clinics and health fairs were frequent mobile phone users, including patients who had advanced ages, significant chronic disease burden, limited educational attainment, and indigenous ethnicity. We found that patients were willing to enroll in a program of m-health self-care support and were able to successfully engage with the program, providing potentially valuable health and self-management information to their healthcare team. Patients’ probability of reporting good, very good, or excellent health via IVR increased substantially over the 12 weeks of their participation. While we cannot assert that these improvements were causally related to program participation, we did find that when patients had more positive health reports, they also were more likely to report that they were taking their medication exactly as prescribed. Medication adherence was a primary focus of the educational messages in the IVR calls.

More generally, this project represents a successful case study for how international research projects focused on m-health can be collaboratively developed with low-resource countries. Despite unreliable Internet access in Bolivia, we were able to establish a trans-national team of investigators and computer engineers, including m-health researchers at the University of Michigan, two units within a major university in La Paz (UCB), the regional governmental lead for NCD policy-making and service delivery (SEDES), and four hospitals from which NCD patients were recruited. Through this and prior projects conducted in Honduras and Mexico, we have identified a variety of strategies for transferring m-health technology internationally that may help these initiatives become more scalable and robust elements of NCD management programs in LMICs.

### Lessons learned

In this project, IVR calls were only available in Spanish. However, 37% of survey respondents with diabetes or hypertension reported speaking a non-Spanish language in the home, and an estimated 35% of people in Bolivia speak the indigenous languages of Aymara or Quechua (45). Without translating programs into the language with which patients are most comfortable, we may be missing opportunities to have a meaningful impact on their NCD self-care. Study participants identifying as Aymara or Quenchua speakers were all able to complete the survey in Spanish. However, this language difference may have introduced some inaccuracies in their responses. Moreover, we found that some indigenous women were skeptical about the study and greater inclusion of surveyors from these communities could be a useful strategy to increase their representation and engagement.

We found that during the enrollment process, older adults tended to have greater difficulties understanding how to use the IVR system and blood pressure home monitoring device. Family members often accompanied these patients and assisted them in asking questions. Inclusion of younger family members in the recruitment process was helpful, although as indicated by Figure 1, barriers still existed in older adults’ program engagement. Greater involvement of family caregivers may be useful for increasing program participation, completion of frequent m-health contacts, and the accuracy of patient reported health and self-care information.

Occasionally, patients reported difficulties completing the IVR calls, because computer server or connectivity problems affected the system in general, or technical issues limited to specific cell phone carriers posed a barrier to call completion. Specific subgroups of patients had unique difficulties, e.g., difficulty with hearing or comprehension among older adults. To understand these barriers and how they could be addressed, we followed up with patients, via phone or in-person meetings. We found that system-related problems were often fixable, in part because the IVR platform was designed to generate detailed reports on the calling process, flagging activity that was out of the ordinary. As novel m-health services are scaled up, a comprehensive and responsive program of technical assistance will be important to encourage enrollment and discourage attrition. For example, it would be beneficial to establish a toll-free help line for patients who have questions or suggestions for improving the service, something that was not done in the current study. Also, completion of a test call upon enrollment may ensure technical functionality and patients’ understanding of the system.

Universities such as UCB typically do not have the infrastructure for scaling and maintaining large m-health programs. For programs such as this one to be sustainable in LMICs, it will be important to either engage cell phone providers and/or build technical capacity through government agencies charged with addressing NCD management. In this ongoing program in Bolivia, conflicting financial incentives and differences in organizational cultural between public and private organizations have continued to be challenging in efforts to bring programs such as this one to scale.

Patients generated clinical alerts during more than half of the weeks in which they completed an IVR call. High rates of alerts may reflect pent-up need for care associated with barriers to accessing NCD treatment and self-management support. Nevertheless, alerts generated at this rate likely would make a service such as this one unacceptable to outpatient teams in LMICs who typically have limited resources even for managing in-person visits. It may be that alert rates would decrease over time as patients learn new self-management skills by participating in the m-health program. Also, it may be that some alerts were generated erroneously because of patients’ limited levels of educational attainment and lack of familiarity with the IVR calls or home blood pressure monitoring. If the latter is true, more time spent in initial training could pay off yielding lower alert rates. Finally, in an environment in which patients may perceive that they have very limited access to clinicians, some patients may use the m-health service as a “call button,” intentionally over reporting health problems in the hopes of speaking directly with their physician for follow-up. Additional research will be important to understand the reasons for high alert rates and how m-health systems (as well as training programs for users) can be designed to keep clinician alerts from over-taxing scarce human resources while improving patients’ access to between-visit support.

### Other issues

As shown in Figure 2, 46% of patients who reported good, very good, or excellent health at baseline reported fair or poor health at least once during their IVR follow-up. This highlights two potential benefits of m-health monitoring. First, several studies have shown that patients may under-report health problems (46) as well as potentially stigmatizing behaviors during in-person interviews when compared to automated assessments. Identifying these problems via IVR may serve to more effectively focus clinicians’ attention on patients who need assistance to prevent worsening health. Also, chronic diseases often have a waxing and waning course with important changes in patients’ symptoms and physiological risk factors occurring in potentially unpredictable ways. For that reason, it is perhaps not surprising that patients reporting very good health when visiting ambulatory care might be in poor health weeks later. The current study suggests that this may be fairly common among patients with diabetes and hypertension in LMICs, and that regular between-visit follow-up via IVR or other m-health tools could be useful to catch emerging health problems before they become acute.

In future work, we plan to continue to expand the clinical foci of these m-health interventions in Bolivia, including other chronic health conditions that are priorities for LMICs. For example, mental health disorders account for a greater share of the global disease burden than HIV/AIDS, tuberculosis, or diabetes; (47) and depression is the second greatest contributor to disability worldwide (48–52). m-Health programs may facilitate systematic monitoring of patients’ depressive symptoms, promptly identifying clinically significant events, and providing tailored psychoeducation (53–56). We currently are working to install new modules as part of the Bolivian IVR platform that address the needs of patients with depression, so that the Bolivian Ministry of Mental Health can evaluate their potential utility for improving mental health service delivery and preventing suicide (53–56). While establishing an independent m-health service in Bolivia is important, it still falls short of the type of international platform required so that m-health knowledge and computing resources can be widely shared across multiple LMICs. We currently are exploring options for establishing that international platform using networks such as the Virtual Campus for Public Health (VCPH, see http://www.campusvirtualsp.org). Twelve Latin American countries participate in the VCPH, and it includes an array of informatics tools for creating and disseminating knowledge bases using Moodle (Modular Object-Oriented Dynamic Learning Environment), an open-source platform used in 235 countries.

### Limitations

We were unable to definitively measure the impact of the m-health service on patients’ health, since the study was not a randomized trial and had only a 12-week follow-up. Feedback on participants’ satisfaction and perceptions about the program was limited to semi-structured telephone interviews with 20 IVR pilot study participants. As a consequence, we are limited in our ability to evaluate the intervention’s impact on subgroups of service users or on important outcomes such as patients’ self-care or health service use. These interviews also were not recorded, transcribed, or analyzed using rigorous qualitative methods, and potential positive feedback or concerns may have been missed. However, two research assistants took detailed notes on each interview, limiting the possibility for missed comments during those conversations. Participants with hypertension were given home blood pressure monitors, and while this may be important to consider as a way to merge m-health messaging with impactful self-care tools, home monitors are rarely available currently in LMICs. No single study can establish the feasibility or utility of m-health programs given the variability of LMICs worldwide. Rather, m-health trials will continue to need evidence for their effectiveness in multiple locations that represent the vast diversity of patients, health systems, and capacity for sustaining large m-health services.

## Conclusion

With these caveats, the present study provides important evidence regarding the feasibility and potential benefit of establishing an independent m-health service in an LMIC. Through active partnerships with leaders in the target country, international collaborators can develop and implement programs for m-health management of chronic diseases. Patients in LMICs who have limited education and diverse socio-ethnic backgrounds can engage successfully with new m-health programs. NCD patients in LMICs can report reliable information during IVR monitoring and self-care support calls, and there is some evidence that such calls can improve their health status. Researchers and policy-makers should continue to explore options for establishing internationally accessible platforms for disseminating knowledge and technical resources for establishing m-health solutions in LMICs.

## Conflict of Interest Statement

The authors declare that the research was conducted in the absence of any commercial or financial relationships that could be construed as a potential conflict of interest.

## Supplementary Material

The Supplementary Material for this article can be found online at http://www.frontiersin.org/Journal/10.3389/fpubh.2014.00095/abstract
