# Supplementary material for: Establishing an Independent Mobile Health Program for Chronic Disease Self-Management Support in Bolivia
Source: Front Public Health. 2014 Aug 13;2:95. doi: 10.3389/fpubh.2014.00095 (PMC4131690; doi:10.3389/fpubh.2014.00095)
Supplement: Supplementary file 1 [file Data_Sheet1.DOC]

# Interactive Voice Response (IVR) Script

**Topic: Diabetes and Hypertension**

**Program: CarePartners**

**NOT FOR REPRODUCTION**

**OR USE WITHOUT PERMISSION**

**FROM DR. JOHN D. PIETTE**

**e:** [**jpiette@umich.edu**](mailto:jpiette@umich.edu)

**Enhancing Caregiver Support Among Spanish-Speaking Patients with Hypertension or Diabetes**

***IVR Caller Validation***

| **ENGLISH** |
| --- |
| **INTROAnsweringMachineOrPerson**  Hello, this is the CarePartner Program. We are calling for [patient name]. [He/she] is expecting my call. Please press 1 to continue.  [If 1 is pressed, go to INTRODMIsCalling]  [After 5 second pause if 1 is not pressed, GO TO INTROAnswerMachineDetected] |
| **INTRODMIsCalling**  Thank you.  [Go To INTROWhoAreYou] |
| **INTROAnswerMachineDetected**  Hello, this is the CarePartner Program. I am sorry we missed you. We will try calling back again at your next scheduled time. Thank you. Good-bye.  [End-call] |
| **INTROWhoAreYou**  If you are the participant, please press “1.”  If you are not the participant, but can bring them to the phone, please press “2.”  If the participant cannot come to the phone at this time, please press “3.”  If this message has reached you in error, or this person cannot be reached at this number, please press “4.”  [IF * REPEAT]  [IF 1, GO TO INTROEnterBirthYear]  [IF 2, GO TO INTROHoldMusic]  [IF 3, GO TO INTROWillCallLater]  [IF 4, GO TO INTROWrongNumber]  [IF INVALID INPUT GO TO DMInvalidResponse and reprompt] |
| ENDCall  Thank you for your time. I’m going to hang up now. Goodbye.  [END CALL] |
| **INTROWhoAreWe**  Thank you. I am calling from the CarePartner program. Today, you will be asked questions about your overall health, about any symptoms you may be having related to your diabetes and about the medicine you are currently taking. You can answer by pressing the numbers on your telephone keypad. The questions will take about 5 to 10 minutes to answer. I am going to ask you each question and then offer possible answer choices. Please choose the response that you think best answers each question.  [IF * REPEAT]  [IF there is a message from CarePartner AND there is a message from the care manager, GO TO INTROBothMsgAvail]  [IF there is NOT a message from the care manager but there is a message from the CarePartner, GO TO INTROCareMsgAvail]  [IF there is NOT a message from CarePartner but there is a message from the care manager, GO TO INTROCareMgnrMsgAvail]  [IF there is NOT a message from either, GO TO INTROStarRepeatPoundSkip] |
| **INTROHoldMusic**  Thank you. The system will wait 5 minutes while you bring the participant to the phone. When you return, press any key to continue.  [PAUSE , THEN GO TO INTROPressAnyKey] |
| **INTROPressAnyKey** (append this to the end of the music file make the total 10 seconds long)  Please press any key to continue.  [ON KeyPress GO TO INTRODMIsCalling]  [REPEAT EVERY 10 SECONDS FOR 5 MINUTES]  [ON TIMEOUT (5 MIN) END CALL] |
| **INTROStarRepeatPoundSkip**  If you need a question repeated, press the star key on your telephone keypad. Please answer all questions so that we can obtain complete information about your health. If you would like to skip the informational feedback and move on to the next question, press the pound key on your telephone keypad at any time.  [IF * REPEAT]  [[IF # SKIP]  [IF 1 GOTO INTROWhoAreWe]  [IF 2 GOTO INTROFirstQuestion] |
| **INTROWillCallLater**  Thank you for your help. We will try to reach [patient first name] at another time. Goodbye.  [END CALL] |
| **DMInvalidResponse**  I am sorry. I do not understand your response. Please listen to the choices, and try again.  [GO TO INTRoWhoAreYou] |
| **INTROWrongNumber**  I am sorry for bothering you. I will not call again. Goodbye.  [END CALL] |
| **INTROFirstQuestion**  Now let’s begin with your first question.  [GO TO GLOBAL HEALTH: GHHowAreYouToday] |

***IVR Global Health***

| **GHHowAreYouToday**  Thinking about your overall health, how are you feeling today?  If you think your overall health is excellent, press “1”.  If you think it’s very good, press “2”.  If you think it’s good, press “3”.  If you think it’s fair, press “4”,  and if you think your overall health is poor, press “5”.  [IF * REPEAT]  [ IF 1,2,3,4,or 5 GO TO GHCompareToLastWeek ]  [IF INVALID GO TO DMInvalidResponse and re-prompt] |
| --- |
| **GHInvalidResponse**  I am sorry. I did not understand your response. Please listen to the choices, and try again.  [RETURN TO SENDING MODULE] |
| **GHCompareToLastWeek**  In terms of your overall health, how would you say that you are feeling this week compared with last week?  If you feel that your overall health is about the same as last week, press “1”.  If you think that it’s better than last week, press “2”.  If you think that it’s worse, press “3”.  [IF * REPEAT]  [IF 1, GO TO *GHCompareSame*]  [IF 2, GO TO *GHCompareBetter*]  [IF 3, GO TO *GHCompareWorse*]  [IF 3, GO TO CEFCompareWorseMsg]  [IF INVALID INPUT GO TO DMInvalidResponse and re-prompt] |
| ***GHCompareSame***  Thank you. Now I’d like to ask about some of the symptoms you may be having related to your diabetes.  [GO TO BedDays] |
| **GHCompareBetter**  I’m glad that you are feeling better this week. Now let’s take a closer look at some of the symptoms you may have been having this past week.  [IF * REPEAT]  [If # SKIP]  [GO TO BedDays] |
| **GHCompareWorse**  I am sorry that you are feeling worse this week. Now let’s take a closer look at some of the symptoms that could be causing you to feel worse.  [IF * REPEAT]  [If # SKIP]  [GO TO BedDays] |
| RXAsPrescribedBP  [Ask this question only if patient is on BP medication]  Now I would like to ask a few questions related to the medicine for your diabetes. Many people have difficulty taking their medicine exactly as prescribed by their doctor.  How often would you say you took your blood pressure medicine exactly as prescribed this past week?  If you always took your blood pressure medicine exactly as prescribed, please press “1”, if you took them exactly as prescribed most of the time,  press “2”, if you sometimes took them as prescribed, press “3”, and if you rarely or never took your medicine exactly as prescribed, please press “4”.  Press the star (*) key to repeat this question.  [IF * REPEAT]  [IF 1, GO TO RXHaveTwoWeekSupplyBP ]  [IF 2,3,4 GO TO RXTakeYourMedsBP ]  [ELSE GO TO DMInvalidResponse and reprompt ] |
| RXTakeYourMedsBP  Although it can be difficult, it is important to take your blood pressure medicine exactly as prescribed. If you are experiencing serious side effects due to your medicine, contact your doctor as soon as possible to talk about whether a change in your medicine may help you feel better. If you don’t have one already, you might consider buying a daily pill dispenser to help you keep track of when you should take your pills.  [IF * REPEAT]  [IF # SKIP FEEDBACK]  [GO TO RXHaveTwoWeekSupplyBP] |
| RXHaveTwoWeekSupplyBP  Do you have enough of your blood pressure medicine to last two weeks? If yes press “1”. If no press “2”.  [IF * REPEAT]  [ IF 1, GO TO RXFillEarlyBP ]  [ IF 2, GO TO RXCallPharmacyBP ]  [ ELSE GO TO DMInvalidResponse and reprompt ]  [If 2, and RXHaveTwoWeekSupplyDB=2, GO TO CEFMedNeedRefill] |
| RXFillEarlyBP  That is great. It is very important that you continue taking your blood pressure medicine every day and never run out. Please continue to fill this prescription early so that you always have the medicine you need.  [GO TO CheckFeet] |
| RXCallPharmacyBP  It is very important that you continue taking your blood pressure medicine every day and never run out. Please call your pharmacy right away for a refill. If you need a new prescription, contact your doctor as soon as possible. I’ll give you your doctor’s phone number at the end of this call.  [GO TO CheckFeet] |

Blood Pressure

| **BPCheck**  (Ask this question only of patients who have endorsed having BP cuffs on the enrollment screen)  During the past week, did you or someone else check your blood pressure on at least 3 different days?  If yes, press "1".  If no, press "2".  [IF * REPEAT]  [IF 1, GO TO SystolicHigh]  [IF 2, GO TO BPCheck_K2] |
| --- |
| **BPCheck _K2**  For a person with diabetes and hypertension like you, it is important to check your blood pressure regularly. You or someone else should try to check it every other day. If you don't check it as often as you should, consider checking your blood pressure at the same time of day every time you check it. For instance, try checking it just before your evening meal. This way, you will get into the habit of checking it regularly.  [GO TO LowSaltDiet] |
| **SystolicHigh**  That's great. During the past week, when you checked your blood pressure, was the top number more than 130 at least half of the time?  During the past week, if your top number was more than 130 at least half of the time, press "1".  If not, press "2".  [IF * REPEAT]  [IF 1, GO TO SystolicHigh_K1)  [(IF 2, GO TO SystolicHigh_K2]  [If 1, GO TO CEFBPMoreThan130] |
| **SystolicHigh_K1**  Your blood pressure may be too high. Even blood pressure that is just a little higher than normal can worsen the complications of diabetes. If you cut back on your salt intake, you may be able to get your blood pressure level down. However, many people need to adjust their medication to bring their blood pressure in line. It is important that you make an appointment with your doctor soon so that you can tell your doctor that your blood pressure has been running higher than normal.  [GO TO SystolicLow] |
| **SystolicHigh_K2**  That's great. Even though your blood pressure has not been running high, it is still important to continue to check it regularly. You should try to check it everyday. Checking your blood pressure at the same time of day -- for instance, just before your evening meal -- may be a good way to get into the habit of checking it. Often there are no symptoms associated with high blood pressure and the only way that you will know that your blood pressure is running high is by checking it.  [GO TO SystolicLow] |
| **SystolicLow**  When you checked your blood pressure during the past week, was the top number less than 100 on two or more days?  During the past week, if your top number was less than 100 on two or more days, press "1".  If not, press "2".  [IF * REPEAT]  [IF 1, GO TO SystolicLow_K1]  [IF 2, GO TO SystolicLow_K2]  [If 1, GO TO CEFBPLessThan100] |
| **SystolicLow_K1**  Your blood pressure may be low. If your blood pressure goes too low, you may get dizzy, fall down, and could hurt yourself. Your medicine may need to be adjusted by your doctor. It is important that you make an appointment with your doctor soon so that you can tell your doctor that your blood pressure has been running lower than normal.  [GO TO LowSaltDiet] |
| **SystolicLow_K2**  That's great. Although your blood pressure has not been running low, continue to check your blood pressure regularly so you will know if it is running low. Try to check it every other day at the same time of day -- for instance, just before your evening meal. This may be a good way to get into the habit of checking it.  [GO TO LowSaltDiet] |
| **LowSaltDiet**  Now I'd like to ask you a question about the foods you eat. During the past week, have you been eating foods that contain a lot of salt? This would include foods such as canned soups, sandwiches with cold cuts, frozen dinners or TV dinners, tortilla chips or canned refried beans.  If you ate a lot of foods that contain salt, press "1".  If not, press "2".  [IF * REPEAT]  [IF 1, GO TO LowSaltDiet_K1]  [IF 2, GO TO LowSaltDiet_K2]  [If 1, GO TO CEFBPHighSalt] |
| **LowSaltDiet_K1**  For a person with diabetes like you, eating salty foods can result in high blood pressure and may also lead to heart disease and kidney failure. There are a few things that you can do right now to lower the amount of salt in your diet. For instance, you can remove extra salt at the table. You can also try to avoid foods with a lot of salt like cold cuts, ham, bacon, cheese, canned soups, canned vegetables, tortilla chips or refried beans. Although it can be difficult to pass up some high salt foods that taste good, eating healthy can help you feel better and prevent more serious health problems.  [GO TO RXAsPrescribedBP] |
| **LowSaltDiet_K2**  Good job on avoiding high salt foods. Eating a low-salt diet is important for a person with diabetes like you.  [GO TO RXAsPrescribedBP] |
| RXAsPrescribedBP  [Ask this question only if patient is on BP medication]  Many people have difficulty taking their medicine exactly as prescribed by their doctor.  How often would you say you took your blood pressure medicine exactly as prescribed this past week?  If you always took your blood pressure medicine exactly as prescribed, please press “1”, if you took them exactly as prescribed most of the time,  press “2”, if you sometimes took them as prescribed, press “3”, and if you rarely or never took your medicine exactly as prescribed, please press “4”.  Press the star (*) key to repeat this question.  [IF * REPEAT]  [IF 1, GO TO RXHaveTwoWeekSupplyBP ]  [IF 2,3,4 GO TO RXTakeYourMedsBP ]  [ELSE GO TO DMInvalidResponse and reprompt ] |
| RXTakeYourMedsBP  Although it can be difficult, it is important to take your blood pressure medicine exactly as prescribed. If you are experiencing serious side effects due to your medicine, contact your doctor as soon as possible to talk about whether a change in your medicine may help you feel better. If you don’t have one already, you might consider buying a daily pill dispenser to help you keep track of when you should take your pills.  [IF * REPEAT]  [IF # SKIP FEEDBACK]  [GO TO RXHaveTwoWeekSupplyBP] |
| RXHaveTwoWeekSupplyBP  Do you have enough of your blood pressure medicine to last two weeks? If yes press “1”. If no press “2”.  [IF * REPEAT]  [ IF 1, GO TO RXFillEarlyBP ]  [ IF 2, GO TO RXCallPharmacyBP ]  [ ELSE GO TO DMInvalidResponse and reprompt ]  [If 2, and RXHaveTwoWeekSupplyDB=2, GO TO CEFMedNeedRefill] |
| RXFillEarlyBP  That is great. It is very important that you continue taking your blood pressure medicine every day and never run out. Please continue to fill this prescription early so that you always have the medicine you need.  [GO TO CheckFeet] |
| RXCallPharmacyBP  It is very important that you continue taking your blood pressure medicine every day and never run out. Please call your pharmacy right away for a refill. If you need a new prescription, contact your doctor as soon as possible. I’ll give you your doctor’s phone number at the end of this call.  [GO TO CheckFeet] |
| **Repeat**  This message will be repeated. |

***IVR Standard Conclusion***

| CONThankYou  That completes this week’s call. Thank you again for participating in this program. We hope it will help you stay as healthy as possible and will help your CarePartner keep track of how you are doing. Even if you don’t find these calls helpful, the information you are providing is very important and will help us understand what works for other people with diabetes who might want more support between visits to their doctor. I will be calling next week at the scheduled time.  [GO TO CONQuestionOrChange] |
| --- |
| CONQuestionOrChange  If you would like to call the CarePartner team with a question about these recorded messages or to change your scheduled calling time, press “1” now.  If you do not need to contact the CarePartner team, press “2.”  [IF * REPEAT]  [IF 1, GO TO *CONContactUsAt*  ]  [IF 2, GO TO CONContactYourDoctor ]  [IF INVALID RESPONSE THEN GO TO CHFInvalidResponse and reprompt ] |
| CONContactUsAt    The CarePartner team can be reached during normal business hours. If you are calling on the evenings or weekends, just leave a message and we’ll get back to you as soon as possible. Our number is: [TTS]  If you would like this number repeated, press”1”.  Otherwise, press “2”.  [ IF 1 GO TO *CONContactUsAt*  ]  [ IF 2, GO TO CONContactYourDoctor ]  [IF INVALID RESPONSE THEN GO TO DMInvalidResponse and reprompt ] |
| CONContactYourDoctor  Remember, the person who knows the most about your health is you.    If you have a question about how to manage your illness, contact your main doctor soon. If you are experiencing any serious problems, call 911 as soon as possible.  If you would like to hear your doctor’s number, press “1”,  otherwise, press “2”.  [IF * REPEAT]  [IF 1, GO TO CONContactDoctorNumber]  [IF 2, GO TO CONNextWeek ]  [IF INVALID RESPONSE THEN GO TO DMInvalidResponse and reprompt ] |
| CONContactDoctorNumber  Your main doctor’s number is XXX-XXXX.  If you would like this number repeated, press “1”.  Otherwise, press “2”.  [Phone number will be filled in by system website]  [IF 1, GO TO CONContactDoctorNumber]  [IF 2, GO TO CONNextWeek] |
| CONNextWeek  Thank you again for your time, and I will call you next week. Goodbye.  [HANG UP] |

| **SPANISH** |
| --- |
| **INTROAnsweringMachineOrPerson**  Hola, este es el Programa CarePartner. Le estamos llamando para [hacer una pausa de tres segundos]. El participante está esperando mi llamada. Por favor oprima 1 para continuar.  [If 1 is pressed, go to INTRODMIsCalling]  [After 5 second pause if 1 is not pressed, GO TO INTROAnswerMachineDetected] |
| **INTRODMIsCalling**  Muchas gracias.  [Go To INTROWhoAreYou] |
| **INTROAnswerMachineDetected**  Hola, este es el Programa CarePartner. Lamentamos no haber podido ponernos en contacto con usted. Trataremos de llamar nuevamente el día y hora de la próxima llamada programada para usted. Muchas gracias. Adiós.  [End-call] |
| **INTROWhoAreYou**  Si usted es el / la participante, por favor oprima “1.” Si usted no es el / la participante, pero puede hacer que él / ella se acerque al teléfono, por favor oprima “2.” Si el / la participante no se puede acercar al teléfono en este momento, por favor oprima “3.” Si usted ha recibido este mensaje por error o si esta persona no se encuentra en este número de teléfono, por favor oprima “4.”  [IF * REPEAT]  [IF 1, GO TO INTROEnterBirthYear]  [IF 2, GO TO INTROHoldMusic]  [IF 3, GO TO INTROWillCallLater]  [IF 4, GO TO INTROWrongNumber]  [IF INVALID INPUT GO TO DMInvalidResponse and reprompt] |
| **ENDCall**  Muchas gracias por su tiempo. Ahora voy a colgar. Adiós.  [END CALL] |
| **INTROWhoAreWe**  Muchas gracias. Le estoy llamando del programa CarePartner. Hoy, a usted se le van a hacer preguntas relativas a su salud en general, acerca de algún síntoma que pudiera estar teniendo en relación con su diabetes y sobre las medicinas que está tomando actualmente. Usted puede responder oprimiendo los números del teclado de su teléfono. Serán necesarios de 5 a 10 minutos para responder a todas las preguntas. Yo le voy a ir haciendo cada pregunta y luego le voy a ofrecer opciones de posibles respuestas. Por favor elija la respuesta que usted considere que es la mejor para cada pregunta.  [IF * REPEAT]  [IF there is a message from CarePartner AND there is a message from the care manager, GO TO INTROBothMsgAvail]  [IF there is NOT a message from the care manager but there is a message from the CarePartner, GO TO INTROCareMsgAvail]  [IF there is NOT a message from CarePartner but there is a message from the care manager, GO TO INTROCareMgnrMsgAvail]  [IF there is NOT a message from either, GO TO INTROStarRepeatPoundSkip] |
| **INTROHoldMusic**  Muchas gracias. El sistema esperará 5 minutos mientras usted acerca al / a la participante al teléfono. Cuando regrese, oprima cualquier tecla para continuar.  [PAUSE , THEN GO TO INTROPressAnyKey] |
| **INTROPressAnyKey** (append this to the end of the music file make the total 10 seconds long)  Por favor oprima cualquier tecla para continuar.  [ON KeyPress GO TO INTRODMIsCalling]  [REPEAT EVERY 10 SECONDS FOR 5 MINUTES]  [ON TIMEOUT (5 MIN) END CALL] |
| **INTROStarRepeatPoundSkip**  Si necesita que se le repita alguna pregunta, oprima la tecla asterisco en el teclado de su teléfono. Por favor responda a todas las preguntas de modo que podamos obtener información completa acerca de su salud. Si usted quisiera omitir la parte correspondiente a la información de retorno y desea pasar a la siguiente pregunta, oprima la tecla numeral en el teclado de su teléfono en cualquier momento.  [IF * REPEAT]  [[IF # SKIP]  [IF 1 GOTO INTROWhoAreWe]  [IF 2 GOTO INTROFirstQuestion] |
| **INTROWillCallLater**  Muchas gracias por su ayuda. Trataremos de ponernos en contacto con [haga una pausa de tres segundos] en otro momento. Adiós.  [END CALL] |
| **DMInvalidResponse**  Lo siento. No entiendo su respuesta. Por favor escuche las opciones y pruebe otra vez.  [GO TO INTRoWhoAreYou] |
| **INTROWrongNumber**  Lamento haberle molestado. No le llamaré más. Adiós.  [END CALL] |
| **INTROFirstQuestion**  Ahora empecemos con su primera pregunta.  [GO TO GLOBAL HEALTH: GHHowAreYouToday] |
| CONThankYou  Esto completa la llamada de esta semana. Gracias otra vez por su participación en este programa. Esperamos que la misma le ayude a mantenerse gozando de tan buena salud como sea posible y también ayudará a su CarePartner a mantenerse al tanto de cómo se encuentra usted. Aún cuando usted no piense que estas llamadas son de ayuda, la información que usted está suministrando es muy importante y la misma nos ayudará a entender mejor qué es lo que funciona para otras personas que podrían querer tener más apoyo entre sus visitas a su médico clínico. Yo voy a llamar la semana próxima a la hora programada.  [GO TO CONQuestionOrChange] |
| CONQuestionOrChange  Si usted quisiera llamar al equipo de CarePartner para hacerle una pregunta sobre estos mensajes grabados o para cambiar el horario de las llamadas programadas, oprima “1” ahora. Si usted no necesita ponerse en contacto con el equipo de CarePartner, oprima “2.”  [IF * REPEAT]  [IF 1, GO TO *CONContactUsAt*  ]  [IF 2, GO TO CONContactYourDoctor ]  [IF INVALID RESPONSE THEN GO TO CHFInvalidResponse and reprompt ] |
| CONContactUsAt    Es posible ponerse en contacto con el equipo del estudio durante las horas normales de trabajo. Si usted va a llamar por las noches o durante los fines de semana, sólo deje un mensaje y le llamaremos de vuelta tan pronto como sea posible. Nuestro número es: [TTS]. Si usted quisiera que se repitiera este número, oprima ”1”. En caso contrario, oprima "2".  [ IF 1 GO TO *CONContactUsAt*  ]  [ IF 2, GO TO CONContactYourDoctor ]  [IF INVALID RESPONSE THEN GO TO DMInvalidResponse and reprompt ] |
| CONContactYourDoctor  Recuerde, la persona que sabe más acerca de su salud, es usted. Si usted tiene alguna pregunta sobre cómo manejar su enfermedad, póngase en contacto con su médico principal en breve. Si usted está experimentando problemas serios, llame al 911 tan pronto como sea posible.  Si usted quisiera escuchar el número de su médico, oprima “1”,  En caso contrario, oprima "2".  [IF * REPEAT]  [IF 1, GO TO CONContactDoctorNumber]  [IF 2, GO TO CONNextWeek ]  [IF INVALID RESPONSE THEN GO TO DMInvalidResponse and reprompt ] |
| CONContactDoctorNumber    El número de su médico principal es [pausa durante tres segundos]  Si usted quisiera que se repitiera este número, oprima ”1”.  En caso contrario, oprima "2".  [Phone number will be filled in by system website]  [IF 1, GO TO CONContactDoctorNumber]  [IF 2, GO TO CONNextWeek] |
| CONNextWeek  Gracias nuevamente por su tiempo, y le llamaré la semana próxima. Adiós.  [HANG UP] |
| CONThankYou  Esto completa la llamada de esta semana. Gracias otra vez por su participación en este programa. Esperamos que la misma le ayude a mantenerse gozando de tan buena salud como sea posible y también ayudará a su CarePartner a mantenerse al tanto de cómo se encuentra usted. Aún cuando usted no piense que estas llamadas son de ayuda, la información que usted está suministrando es muy importante y la misma nos ayudará a entender mejor qué es lo que funciona para otras personas que podrían querer tener más apoyo entre sus visitas a su médico clínico. Yo voy a llamar la semana próxima a la hora programada.  [GO TO CONQuestionOrChange] |
| CONQuestionOrChange  Si usted quisiera llamar al equipo de CarePartner para hacerle una pregunta sobre estos mensajes grabados o para cambiar el horario de las llamadas programadas, oprima “1” ahora. Si usted no necesita ponerse en contacto con el equipo de CarePartner, oprima “2.”  [IF * REPEAT]  [IF 1, GO TO *CONContactUsAt*  ]  [IF 2, GO TO CONContactYourDoctor ]  [IF INVALID RESPONSE THEN GO TO CHFInvalidResponse and reprompt ] |
| CONContactUsAt    Es posible ponerse en contacto con el equipo del estudio durante las horas normales de trabajo. Si usted va a llamar por las noches o durante los fines de semana, sólo deje un mensaje y le llamaremos de vuelta tan pronto como sea posible. Nuestro número es: [TTS]. Si usted quisiera que se repitiera este número, oprima ”1”. En caso contrario, oprima "2".  [ IF 1 GO TO *CONContactUsAt*  ]  [ IF 2, GO TO CONContactYourDoctor ]  [IF INVALID RESPONSE THEN GO TO DMInvalidResponse and reprompt ] |
| CONContactYourDoctor  Recuerde, la persona que sabe más acerca de su salud, es usted. Si usted tiene alguna pregunta sobre cómo manejar su enfermedad, póngase en contacto con su médico principal en breve. Si usted está experimentando problemas serios, llame al 911 tan pronto como sea posible.  Si usted quisiera escuchar el número de su médico, oprima “1”,  En caso contrario, oprima "2".  [IF * REPEAT]  [IF 1, GO TO CONContactDoctorNumber]  [IF 2, GO TO CONNextWeek ]  [IF INVALID RESPONSE THEN GO TO DMInvalidResponse and reprompt ] |
| CONContactDoctorNumber    El número de su médico principal es [pausa durante tres segundos]  Si usted quisiera que se repitiera este número, oprima ”1”.  En caso contrario, oprima "2".  [Phone number will be filled in by system website]  [IF 1, GO TO CONContactDoctorNumber]  [IF 2, GO TO CONNextWeek] |
| CONNextWeek  Gracias nuevamente por su tiempo, y le llamaré la semana próxima. Adiós.  [HANG UP] |
| CONThankYou  Esto completa la llamada de esta semana. Gracias otra vez por su participación en este programa. Esperamos que la misma le ayude a mantenerse gozando de tan buena salud como sea posible y también ayudará a su CarePartner a mantenerse al tanto de cómo se encuentra usted. Aún cuando usted no piense que estas llamadas son de ayuda, la información que usted está suministrando es muy importante y la misma nos ayudará a entender mejor qué es lo que funciona para otras personas que podrían querer tener más apoyo entre sus visitas a su médico clínico. Yo voy a llamar la semana próxima a la hora programada.  [GO TO CONQuestionOrChange] |
| CONQuestionOrChange  Si usted quisiera llamar al equipo de CarePartner para hacerle una pregunta sobre estos mensajes grabados o para cambiar el horario de las llamadas programadas, oprima “1” ahora. Si usted no necesita ponerse en contacto con el equipo de CarePartner, oprima “2.”  [IF * REPEAT]  [IF 1, GO TO *CONContactUsAt*  ]  [IF 2, GO TO CONContactYourDoctor ]  [IF INVALID RESPONSE THEN GO TO CHFInvalidResponse and reprompt ] |
| CONContactUsAt    Es posible ponerse en contacto con el equipo del estudio durante las horas normales de trabajo. Si usted va a llamar por las noches o durante los fines de semana, sólo deje un mensaje y le llamaremos de vuelta tan pronto como sea posible. Nuestro número es: [TTS]. Si usted quisiera que se repitiera este número, oprima ”1”. En caso contrario, oprima "2".  [ IF 1 GO TO *CONContactUsAt*  ]  [ IF 2, GO TO CONContactYourDoctor ]  [IF INVALID RESPONSE THEN GO TO DMInvalidResponse and reprompt ] |
| CONContactYourDoctor  Recuerde, la persona que sabe más acerca de su salud, es usted. Si usted tiene alguna pregunta sobre cómo manejar su enfermedad, póngase en contacto con su médico principal en breve. Si usted está experimentando problemas serios, llame al 911 tan pronto como sea posible.  Si usted quisiera escuchar el número de su médico, oprima “1”,  En caso contrario, oprima "2".  [IF * REPEAT]  [IF 1, GO TO CONContactDoctorNumber]  [IF 2, GO TO CONNextWeek ]  [IF INVALID RESPONSE THEN GO TO DMInvalidResponse and reprompt ] |
| CONContactDoctorNumber    El número de su médico principal es [pausa durante tres segundos]  Si usted quisiera que se repitiera este número, oprima ”1”.  En caso contrario, oprima "2".  [Phone number will be filled in by system website]  [IF 1, GO TO CONContactDoctorNumber]  [IF 2, GO TO CONNextWeek] |
| CONNextWeek  Gracias nuevamente por su tiempo, y le llamaré la semana próxima. Adiós.  [HANG UP] |
| CONThankYou  Esto completa la llamada de esta semana. Gracias otra vez por su participación en este programa. Esperamos que la misma le ayude a mantenerse gozando de tan buena salud como sea posible y también ayudará a su CarePartner a mantenerse al tanto de cómo se encuentra usted. Aún cuando usted no piense que estas llamadas son de ayuda, la información que usted está suministrando es muy importante y la misma nos ayudará a entender mejor qué es lo que funciona para otras personas que podrían querer tener más apoyo entre sus visitas a su médico clínico. Yo voy a llamar la semana próxima a la hora programada.  [GO TO CONQuestionOrChange] |
| CONQuestionOrChange  Si usted quisiera llamar al equipo de CarePartner para hacerle una pregunta sobre estos mensajes grabados o para cambiar el horario de las llamadas programadas, oprima “1” ahora. Si usted no necesita ponerse en contacto con el equipo de CarePartner, oprima “2.”  [IF * REPEAT]  [IF 1, GO TO *CONContactUsAt*  ]  [IF 2, GO TO CONContactYourDoctor ]  [IF INVALID RESPONSE THEN GO TO CHFInvalidResponse and reprompt ] |
| CONContactUsAt    Es posible ponerse en contacto con el equipo del estudio durante las horas normales de trabajo. Si usted va a llamar por las noches o durante los fines de semana, sólo deje un mensaje y le llamaremos de vuelta tan pronto como sea posible. Nuestro número es: [TTS]. Si usted quisiera que se repitiera este número, oprima ”1”. En caso contrario, oprima "2".  [ IF 1 GO TO *CONContactUsAt*  ]  [ IF 2, GO TO CONContactYourDoctor ]  [IF INVALID RESPONSE THEN GO TO DMInvalidResponse and reprompt ] |
| CONContactYourDoctor  Recuerde, la persona que sabe más acerca de su salud, es usted. Si usted tiene alguna pregunta sobre cómo manejar su enfermedad, póngase en contacto con su médico principal en breve. Si usted está experimentando problemas serios, llame al 911 tan pronto como sea posible.  Si usted quisiera escuchar el número de su médico, oprima “1”,  En caso contrario, oprima "2".  [IF * REPEAT]  [IF 1, GO TO CONContactDoctorNumber]  [IF 2, GO TO CONNextWeek ]  [IF INVALID RESPONSE THEN GO TO DMInvalidResponse and reprompt ] |
| CONContactDoctorNumber    El número de su médico principal es [pausa durante tres segundos]  Si usted quisiera que se repitiera este número, oprima ”1”.  En caso contrario, oprima "2".  [Phone number will be filled in by system website]  [IF 1, GO TO CONContactDoctorNumber]  [IF 2, GO TO CONNextWeek] |
| CONNextWeek  Gracias nuevamente por su tiempo, y le llamaré la semana próxima. Adiós.  [HANG UP] |
| CONThankYou  Esto completa la llamada de esta semana. Gracias otra vez por su participación en este programa. Esperamos que la misma le ayude a mantenerse gozando de tan buena salud como sea posible y también ayudará a su CarePartner a mantenerse al tanto de cómo se encuentra usted. Aún cuando usted no piense que estas llamadas son de ayuda, la información que usted está suministrando es muy importante y la misma nos ayudará a entender mejor qué es lo que funciona para otras personas que podrían querer tener más apoyo entre sus visitas a su médico clínico. Yo voy a llamar la semana próxima a la hora programada.  [GO TO CONQuestionOrChange] |
| CONQuestionOrChange  Si usted quisiera llamar al equipo de CarePartner para hacerle una pregunta sobre estos mensajes grabados o para cambiar el horario de las llamadas programadas, oprima “1” ahora. Si usted no necesita ponerse en contacto con el equipo de CarePartner, oprima “2.”  [IF * REPEAT]  [IF 1, GO TO *CONContactUsAt*  ]  [IF 2, GO TO CONContactYourDoctor ]  [IF INVALID RESPONSE THEN GO TO CHFInvalidResponse and reprompt ] |
| CONContactUsAt    Es posible ponerse en contacto con el equipo del estudio durante las horas normales de trabajo. Si usted va a llamar por las noches o durante los fines de semana, sólo deje un mensaje y le llamaremos de vuelta tan pronto como sea posible. Nuestro número es: [TTS]. Si usted quisiera que se repitiera este número, oprima ”1”. En caso contrario, oprima "2".  [ IF 1 GO TO *CONContactUsAt*  ]  [ IF 2, GO TO CONContactYourDoctor ]  [IF INVALID RESPONSE THEN GO TO DMInvalidResponse and reprompt ] |
| CONContactYourDoctor  Recuerde, la persona que sabe más acerca de su salud, es usted. Si usted tiene alguna pregunta sobre cómo manejar su enfermedad, póngase en contacto con su médico principal en breve. Si usted está experimentando problemas serios, llame al 911 tan pronto como sea posible.  Si usted quisiera escuchar el número de su médico, oprima “1”,  En caso contrario, oprima "2".  [IF * REPEAT]  [IF 1, GO TO CONContactDoctorNumber]  [IF 2, GO TO CONNextWeek ]  [IF INVALID RESPONSE THEN GO TO DMInvalidResponse and reprompt ] |
| CONContactDoctorNumber    El número de su médico principal es [pausa durante tres segundos]  Si usted quisiera que se repitiera este número, oprima ”1”.  En caso contrario, oprima "2".  [Phone number will be filled in by system website]  [IF 1, GO TO CONContactDoctorNumber]  [IF 2, GO TO CONNextWeek] |
| CONNextWeek  Gracias nuevamente por su tiempo, y le llamaré la semana próxima. Adiós.  [HANG UP] |
| CONThankYou  Esto completa la llamada de esta semana. Gracias otra vez por su participación en este programa. Esperamos que la misma le ayude a mantenerse gozando de tan buena salud como sea posible y también ayudará a su CarePartner a mantenerse al tanto de cómo se encuentra usted. Aún cuando usted no piense que estas llamadas son de ayuda, la información que usted está suministrando es muy importante y la misma nos ayudará a entender mejor qué es lo que funciona para otras personas que podrían querer tener más apoyo entre sus visitas a su médico clínico. Yo voy a llamar la semana próxima a la hora programada.  [GO TO CONQuestionOrChange] |
| CONQuestionOrChange  Si usted quisiera llamar al equipo de CarePartner para hacerle una pregunta sobre estos mensajes grabados o para cambiar el horario de las llamadas programadas, oprima “1” ahora. Si usted no necesita ponerse en contacto con el equipo de CarePartner, oprima “2.”  [IF * REPEAT]  [IF 1, GO TO *CONContactUsAt*  ]  [IF 2, GO TO CONContactYourDoctor ]  [IF INVALID RESPONSE THEN GO TO CHFInvalidResponse and reprompt ] |
| CONContactUsAt    Es posible ponerse en contacto con el equipo del estudio durante las horas normales de trabajo. Si usted va a llamar por las noches o durante los fines de semana, sólo deje un mensaje y le llamaremos de vuelta tan pronto como sea posible. Nuestro número es: [TTS]. Si usted quisiera que se repitiera este número, oprima ”1”. En caso contrario, oprima "2".  [ IF 1 GO TO *CONContactUsAt*  ]  [ IF 2, GO TO CONContactYourDoctor ]  [IF INVALID RESPONSE THEN GO TO DMInvalidResponse and reprompt ] |
| CONContactYourDoctor  Recuerde, la persona que sabe más acerca de su salud, es usted. Si usted tiene alguna pregunta sobre cómo manejar su enfermedad, póngase en contacto con su médico principal en breve. Si usted está experimentando problemas serios, llame al 911 tan pronto como sea posible.  Si usted quisiera escuchar el número de su médico, oprima “1”,  En caso contrario, oprima "2".  [IF * REPEAT]  [IF 1, GO TO CONContactDoctorNumber]  [IF 2, GO TO CONNextWeek ]  [IF INVALID RESPONSE THEN GO TO DMInvalidResponse and reprompt ] |
| CONContactDoctorNumber    El número de su médico principal es [pausa durante tres segundos]  Si usted quisiera que se repitiera este número, oprima ”1”.  En caso contrario, oprima "2".  [Phone number will be filled in by system website]  [IF 1, GO TO CONContactDoctorNumber]  [IF 2, GO TO CONNextWeek] |
| CONNextWeek  Gracias nuevamente por su tiempo, y le llamaré la semana próxima. Adiós.  [HANG UP] |
